# A Geometric Approach for Real-Time Forward Kinematics of the General Stewart Platform

**DOI:** 10.3390/s22134829

**Published:** 2022-06-26

**Authors:** Fangfang Yang, Xiaojun Tan, Zhe Wang, Zhenfeng Lu, Tao He

**Affiliations:** 1 School of Intelligent Systems Engineering, Sun Yat-sen University, Guangzhou 510275, China; yangff7@mail.sysu.edu.cn (F.Y.); tanxj@mail.sysu.edu.cn (X.T.); luzhf6@mail2.sysu.edu.cn (Z.L.); 2 Department of Advanced Design and Systems Engineering, City University of Hong Kong, Hong Kong; dr.zhe.wang@outlook.com

**Keywords:** the Stewart platform, forward kinematics, geometric approach, real-time

## Abstract

This paper presents a geometric approach for real-time forward kinematics of the general Stewart platform, which consists of two rigid bodies connected by six general serial manipulators. By describing the rigid-body motion as exponential of twist, and taking advantage of the product of exponentials formula, a step-by-step derivation of the proposed algorithm is presented. As the algorithm naturally solves all passive joint displacements, the correctness is then verified by comparing the forward-kinematic solutions from all chains. The convergence ability and robustness of the proposed algorithm are demonstrated with large amounts of numerical simulations. In all test cases, the proposed algorithm terminates within four iterations, converging with near-quadratic speed. Finally, the proposed algorithm is also implemented on a mainstream embedded motion controller. Compared with the incremental method, the proposed algorithm is more robust, with an average execution time of 0.48 ms, meeting the requirements of most applications, such as kinematic calibration, motion simulation, and real-time control.

## 1. Introduction

**Figure 1:**
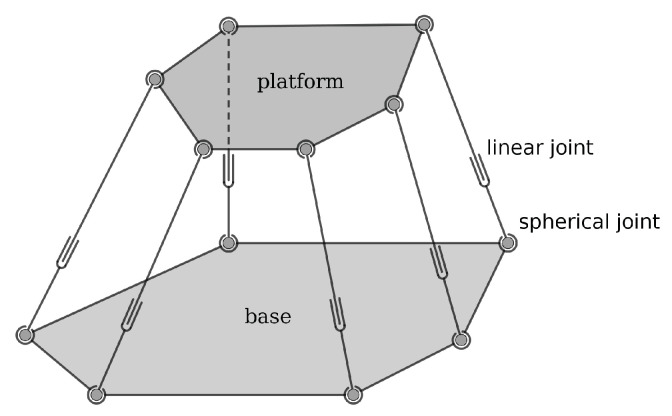
The 6-SPS Stewart platform.

The Stewart platform, originally proposed by D. Stewart in 1965 [1], is one of the most celebrated parallel manipulators. As illustrated in  Figure 1, it has a mounting base B and a moving platform P, connected by six linear actuators via spherical/universal joints. The Stewart platform has wide applications in flight simulation, animatronics, crane technology, underwater research, robotics and so on. Compared with its serial counterparts, the Stewart platform has higher force/torque capacity, greater structure rigidity and superior positioning accuracy. However, the complex coupling of chains also greatly increases the difficulties in forward kinematics, which is essential for both theoretical analysis and practical applications.

For the real-time control of the general Stewart platform, either with optimized design or after kinematic calibration, a fast forward kinematics algorithm must be developed. Denoting $b_{i}$ and $p_{i}$ as the center points of the spherical joints corresponding to the *i*-th chain, the forward kinematics of the Stewart platform is equivalent to solving the following equations [2]:

(1) $$\begin{array}{c} ∥Rp_{i}+T-b_{i}∥=L_{i},\;\mathrm{for}\;i=1,2,\ldots 6, \end{array}$$

where R and T are the rotation matrix and translation vector of the moving platform to be solved, $L_{i}$ is the given length of *i*-th leg.

Existing methods can be generally divided into four categories, analytical methods, numerical methods, extra-sensor methods, and intelligent algorithms. Initially, most researchers focused on finding all possible closed forms by considering special configurations where some of the base or platform spherical joints coincide. Griffis et al. [3] considered the forward kinematics of a 3–3 Stewart platform and obtained an eighth degree polynomial. Lin et al. [4] solved the forward kinematics of the 4–4 case. More difficult cases, such as 5–4 [5,6] and 6–4 [7] Stewart platforms were also solved analytically. The final 6–6 case was independently analysed and solved, such as in Wen et al. [8], Sreenivasan et al. [9], and Dasgupta et al. [10]. Further study of Equation (1) showed that the forward kinematics eventually boiled down to a univariate polynomial of 40-th order [11,12,13]. To actually compute these solutions, a number of root-finding methods were then proposed, including algebraic elimination [14,15,16], interval analysis [17], and continuation [12,18].

Although analytical methods can obtain all possible solutions to forward kinematics, they are sensitive to numerical errors. Moreover, in real applications, an extra procedure must be presented to determine a unique actual pose from all possible solutions. Numerical methods and extra-sensor methods are two common schemes for this purpose. Since forward kinematics essentially boils down to solving non-linear equations, Newton–Raphson iterative algorithms [19,20,21] were adopted extensively for their high computational efficiency and fast convergence speed. Xie et al. [22] formulated the forward kinematics as an unconstrained optimization problem on SE(3) and compared their results with the Euler angle and quaternion parametrization schemes. However, the convergence of Newton–Raphson method depends heavily on the selection of initial values. Yang et al. [23] proposed the global Newton–Raphson method with monotonic descent algorithm to achieve global convergence regardless of the initial guess. Wang [24] tackled the issue by adopting a series of small leg length changes to finally obtain a unique forward kinematic solution. Pratik et al. [25] proposed a hybrid strategy that used a neural network as a bootstrap for the standard Newton–Raphson algorithm to yield a unique solution. The forward kinematics can also be settled with auxillary sensors. To simplify the sophisticated forward kinematics, as well as obtain a unique solution simultaneously, Bonev et al. [26] used three linear sensors connecting the moving platform and the base, Chiu et al. [27] used two rotatory sensors and one displacement sensor, and Cheng et al. [28] used one linear sensor to measure the distance of the moving platform. Although the extra-sensor method is proven effective, it comes with extra economic cost as well as assembly and measurement errors, which significantly limits the application range.

Recently, some researchers had also used machine learning techniques, such as support vector machines [29] and artificial neural network [30,31], to solve the problem. Chauhan et al. [32] proposed soft-computing-based schemes to resolve the forward kinematics of the Stewart platform, in which a neural-network-based function estimator was proposed for the forward kinematics and then trained the network with meta-heuristic optimization procedures to revise the weights and bias values of the neural network. The major problem with learning techniques is that the training process requires an abundant amount of data and usually takes hours for fine-tuning. Moreover, if kinematic calibration is present, the training process has to be done for every machine.

Although a lot of research has been done on the forward kinematics of the 6-SPS, the forward kinematics of the general Stewart platform has not yet been fully explored. Most existing works focused on the standard 6-SPS structure, very few authors considered other variants of the Stewart platform such as 6-RUS [33] and 6-RSS [34]. The so-called general Stewart platform studied in existing works is actually a 6-SPS type platform with non-planar base and moving platform, which is inadequate for applications such as geometric calibration. For the general Stewart platform considered herein, every chain is essentially a general 6-degrees-of-freedom serial manipulator, with planar or non-planar mounting base and moving platform. By general serial manipulator, each joint can be prismatic, revolute, or helical, with no presumed geometric constraints between joints, such as perpendicularity, parallelism, and intersection. From here onwards, the general Stewart platform, if not otherwise stated, adheres to the above definition. Such generalization allows the control of a wide range of modified Stewart platforms designed to fulfil specific optimization criteria or special task requirements, such as in [35,36], where the modified Stewart platform was designed for better dexterity. In [37], a special Stewart platform with non-intersecting U-joint was considered for precision surgery, which clearly violates Equation (1). Moreover, such generation enables the kinematic calibration of the Stewart platform. Wang et al. [38] showed that the accuracy of the Stewart platform can be of the same level as a serial manipulator with the same nominal dimensions owing to manufacturing tolerances. Later, Masory et al. [39] showed that the accuracy of the Stewart platform can be enhanced by at least one order of magnitude after kinematic calibration, for which purpose, an error model of the Stewart platform, which can be regarded as the general Stewart platform, was established.

In this paper, we intend to develop a numerical algorithm for real-time forward kinematics of the general Stewart platform. For this purpose, a geometric approach is adopted for describing rigid body motion. Different from Denavit–Hartenberg and dual-quaternion parametrization, the geometric approach describes rigid body motion via exponential mapping of twist. The product of exponentials (POE) formula is utilized for the forward kinematics of the general serial manipulator. Using a loop closure condition, a very nice and neat differential relationship between task space and joint space are then established, based on which an iterative algorithm is presented. Simulation studies are then carried out to verify the effectiveness and convergence ability of the proposed algorithm. Finally, the algorithm is implemented on an embedded controller to demonstrate its capability in real applications.

The major contributions of this paper are summarized as follows.

1. A geometric algorithm is proposed for real-time forward kinematics of the general Stewart platform, with no geometric constraints, such as perpendicularity, parallelism, and intersection, are presumed between joints. The proposed method is derived with regard to a general Stewart platform, and can be readily applied to other existing spatial manipulators, including but not limited to 6-RUS and 6-RSS manipulators.
2. The proposed algorithm is successfully implemented on an embedded controller. Compared with the incremental method, which only applies to 6-SPS method, the proposed algorithm is more robust, with a comparative execution time of 0.48 ms, which is sufficient for most real-time applications.

The rest of this paper is organized as follows. In Section 2, an introduction to the mathematical foundation of the proposed geometric method is presented, based on which the details of the proposed forward kinematics algorithm is presented in Section 3. To demonstrate the effectiveness of the proposed algorithm, simulations and experiments are presented in Section 4. Finally, Section 5 concludes this paper.

## 2. Notation and Terminology

In this section, we present the mathematical tools for modelling a general serial robot, the notation and motivation presented herein basically follow [40].

### 2.1. Lie Group and Lie Algebra

Mathematically, a Lie group is a group that is also a differentiable manifold. To every Lie group we can associate a Lie algebra whose underlying vector space is the tangent space of the Lie group at the identity element. An elementary introduction to Lie group and Lie algebra can be found in [41].

The collection of all rotation matrices, known as the special orthogonal group SO(3), is a Lie Group. The corresponding Lie algebra, denoted as so(3), is the set of all screw-symmetric 3 × 3 matrices. so(3) and $R^{3}$ are isomorphic via the hat operator ${\left(\;\cdot \;\right)}^{\wedge }:R^{3}\to \mathrm{so}\left(3\right)$, defined as follows:

(2) $$\begin{array}{c} {\left(\;\cdot \;\right)}^{\wedge }:\left[\begin{array}{c} \omega_{1}\\ \omega_{2}\\ \omega_{3} \end{array}\right]\mapsto \left[\begin{array}{ccc} 0 & -\omega_{3} & \omega_{2}\\ \omega_{3} & 0 & -\omega_{1}\\ -\omega_{2} & \omega_{1} & 0 \end{array}\right]. \end{array}$$

Additionally, the inverse of the hat operator is defined as the vee operator ${\left(\;\cdot \;\right)}^{\vee }:\mathrm{so}\left(3\right)\mapsto R^{3}$.

The set of all the rigid body transformations, known as the special Euclidean group SE(3), is also a Lie group. An element g∈SE(3) can be completely described by a 4 × 4 homogeneous matrix:

(3) $$g=\left[\begin{array}{cc} R & P\\ 0 & 1 \end{array}\right]\in R^{4\times 4},$$

where R∈SO(3) denotes the rotation between the two coordinate frames and $P\in R^{3}$ denotes the spatial displacement of the origin. The corresponding Lie algebra se(3) is isomorphic to $R^{6}$, with the hat operator ${\left(\;\cdot \;\right)}^{\wedge }:R^{6}\to \mathrm{se}\left(3\right)$ defined as

(4) $$\begin{array}{c} {\left(\;\cdot \;\right)}^{\wedge }:\left[\begin{array}{c} v\\ \omega \end{array}\right]\mapsto \left[\begin{array}{cc} \hat{\omega } & v\\ 0 & 0 \end{array}\right]. \end{array}$$

Likewise, the inverse of the hat operator is defined as the vee operator ${\left(\;\cdot \;\right)}^{\vee }:\mathrm{se}\left(3\right)\mapsto R^{6}$.

Given a rigid body motion g(t)∈SE(3), the spatial velocity and body velocity are defined as

(5) $$\begin{array}{cc} V_{s} & ={\left(\dot{g}g^{-1}\right)}^{\wedge } \end{array}$$

(6) $$\begin{array}{cc} V_{b} & ={\left(g^{-1}\dot{g}\right)}^{\wedge }. \end{array}$$

The spatial velocity and body velocity are essentially the same thing expressed in different frames, their relationship can be conveniently expressed using the adjoint map associated with *g*, $Ad_{g}\left(\;\cdot \;\right):\mathrm{se}\left(3\right)\to \mathrm{se}\left(3\right)$ defined as

(7) $$\begin{array}{c} {\mathrm{Ad}}_{g}\left(\;\cdot \;\right):\xi \mapsto {\left(g\;\cdot \;\hat{\xi }\;\cdot \;g^{-1}\right)}^{\vee }. \end{array}$$

### 2.2. Exponential Map

For a vector field *X* in tangent space $T_{e}G$ at group identity *e*, there exists a smooth one-parameter subgroup $\gamma_{X}\left(t\right)$ of a Lie group *G* parametrized by a scalar t∈R. There also exists an exponential map defined as

(8) $$\begin{array}{c} \exp:T_{e}G\times R\mapsto G,\left(X,t\right)\mapsto \exp\left(tX\right). \end{array}$$

For SO(3) and SE(3), the exponential map corresponds to the usual matrix exponentiation $\exp\left(\;\cdot \;\right):R^{n\times n}\to R^{n\times n}$,

(9) $$\begin{array}{c} \exp\left(\;\cdot \;\right):M\mapsto \sum_{n=0}^{\infty }\frac{1}{n!}M^{n}. \end{array}$$

## 3. Forward Kinematics of the General Stewart Platform

In this section, armed with the tools presented in Section 2, an algorithm is proposed for the forward kinematics of the general Stewart platform.

### 3.1. Kinematics of General Serial Robot

**Figure 2:**
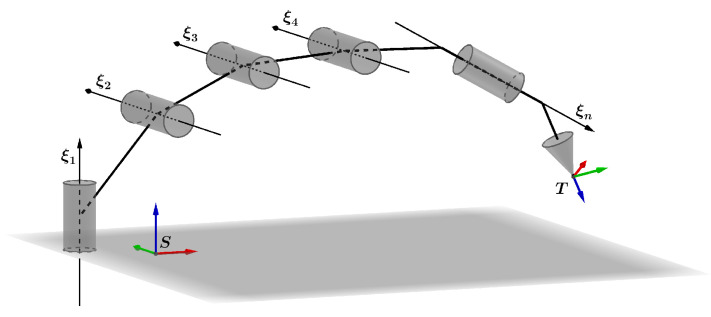
A general serial robot with *n* joints.

For a general serial robot with any number of helical joints, as shown in  Figure 2, usually a spatial frame *S* and a tool frame *T* are attached at the base and the end-effector, respectively. The transformation of tool frame with respect to the spatial frame can be expressed via the POE formula [40]:

(10) $$\begin{array}{c} g_{st}\left(\Theta \right)=e^{{\hat{\xi }}_{1}\theta_{1}}\ldots e^{{\hat{\xi }}_{n}\theta_{n}}g_{st}\left(0\right), \end{array}$$

where $\xi_{i}$ and $\theta_{i}$ are the twist and displacement corresponding to the *i*-th joint, respectively; $g_{st}\left(0\right)$ is the initial rigid displacement between the tool frame and the spatial frame.

The spatial velocity of the tool frame is defined as

(11) $$\begin{array}{c} V_{st}={\left({\dot{g}}_{st}g_{st}^{-1}\right)}^{\vee }=\left[\begin{array}{cccc} \xi_{1} & \xi_{2}^{\prime } & \ldots & \xi_{n}^{\prime } \end{array}\right]{\left[\begin{array}{cc} {\dot{\theta }}_{1} & {\dot{\theta }}_{2}\ldots {\dot{\theta }}_{n} \end{array}\right]}^{T}, \end{array}$$

where

(12) $$\begin{array}{c} \xi_{i}^{\prime }=\left\{\begin{array}{cc} \xi_{1} & i=1,\\ \prod_{k=1}^{i-1}{\mathrm{Ad}}_{e^{{\hat{\xi }}_{k}\theta_{k}}}\xi_{i} & \mathrm{otherwise}. \end{array}\right. \end{array}$$

The spatial Jacobian matrix is defined as

(13) $$\begin{array}{c} J_{st}=\left[\begin{array}{cccc} \xi_{1} & \xi_{2}^{\prime } & \ldots & \xi_{n}^{\prime } \end{array}\right]. \end{array}$$

At this point, it is worth mentioning that when dΘ is small enough,

(14) $$\begin{array}{c} g_{st}\left(\Theta +d\Theta \right)-g_{st}\left(\Theta \right)\approx {\left(J_{st}d\Theta \right)}^{\wedge }g_{st}\left(\Theta \right). \end{array}$$

The relation follows directly from the definition of $V_{st}$, and shall be useful in the following derivations.

### 3.2. The Proposed Algorithm

First of all, consider the *i*-th chain of the general Stewart platform, denote $\xi_{i}^{j}$ and $\theta_{i}^{j}$ twist and joint displacement of the *j*-th joint. According to Equation (10), the forward kinematics can be written as:

(15) $$\begin{array}{c} g_{i}=\prod_{j=1}^{6}e^{{\hat{\xi }}_{i}^{j}\theta_{i}^{j}}g_{i}\left(0\right), \end{array}$$

where $g_{i}\left(0\right)$ is the initial rigid body displacement. It is shown that the minimal number of parameters needed to determine the initial position of joint twists and rigid body displacements is 5h+4r+2t+6 [42,43], where *h* is the number of helical joints, *r* is the number of revolute joints, *t* is the number of prismatic joints, and the constant 6 refers to the number of parameters needed to determine the initial rigid displacement. For instance, the Stewart platform considered later in Section 4 contains 1 helical joint, 4 revolute joints, and 1 prismatic joint in each chain, hence 29 geometric parameters are needed. Subsequently, a set of 174 geometric parameters are needed for all six chains.

At this point, the forward kinematics of the general Stewart platform is equivalent to solving the following equations:

(16) $$\begin{array}{c} g_{i}=g_{st},\;\mathrm{for}\;i=1,\ldots 6, \end{array}$$

where $g_{st}$ is the 4×4 homogeneous matrix representing the configuration of tool frame at a given input to solve. Excluding the actuator of each chain, there are a total of 30 unknown passive joint displacements, meanwhile there are 6 unknowns in $g_{st}$. Hence, a total of 36 unknowns are present. On the other hand, each of the equations provides essentially 6 constraints, so the number of constraints is 36 in general (degenerate in case of singularities). Hence, the system of equations is complete as the number of variables agrees with the number of constraints. To solve the forward kinematics, consider the *m*-th and the *n*-th chains of the general Stewart platform, immediately we have

(17) $$\begin{array}{c} g_{m}=g_{st}=g_{n}, \end{array}$$

where $g_{m}$ and $g_{n}$ denote the forward kinematics of *m*-th and *n*-th chains, respectively. Generally, Equation (17) does not hold at initial joint values. Nonetheless, according to Equation (14), if the joint deviations are small enough, the following approximation holds:

(18) $$\begin{array}{c} \left({\left(J_{m}\delta \Theta_{m}\right)}^{\wedge }+I_{4}\right)g_{m}\approx \left({\left(J_{n}\delta \Theta_{n}\right)}^{\wedge }+I_{4}\right)g_{n}, \end{array}$$

where $J_{m}$ and $J_{n}$ are the spatial Jacobians corresponding to *m*-th chain and *n*-th chain, respectively; $\delta \Theta_{m}$ and $\delta \Theta_{n}$ are the joint deviations corresponding to *m*-th chain and *n*-th chain, respectively. Multiplying both sides with $g_{m}^{-1}$, the following approximation is obtained with some re-arrangements:

(19) $$\begin{array}{c} {\left(J_{m}\Theta_{m}\right)}^{\wedge }-{\left(J_{n}\Theta_{n}\right)}^{\wedge }\left(g_{n}g_{m}^{-1}\right)\approx g_{n}g_{m}^{-1}-I_{4}. \end{array}$$

In case the joint deviations are small enough, the following approximation can be safely used:

(20) $$\begin{array}{c} {\left(J_{n}\Theta_{n}\right)}^{\wedge }g_{n}g_{m}^{-1}\approx {\left(J_{n}\Theta_{n}\right)}^{\wedge }. \end{array}$$

Substitution back into Equation (19) yields

(21) $$\begin{array}{c} {\left(J_{m}\Theta_{m}\right)}^{\wedge }-{\left(J_{n}\Theta_{n}\right)}^{\wedge }\approx g_{n}g_{m}^{-1}-I_{4}, \end{array}$$

which up to the first order is equivalent to

(22) $$\begin{array}{c} J_{m}\delta \Theta_{m}-J_{n}\delta \Theta_{n}\approx \log^{\vee }\left(g_{n}g_{m}^{-1}\right), \end{array}$$

where log:SE(3)↦se(3) is the inverse operator of exp(·), and $\log^{\vee }\left(g_{n}g_{m}^{-1}\right)$ abbreviates for ${\left(\log(g_{n}g_{m}^{-1})\right)}^{\vee }$. To this point, a differential relationship between joint space and task space for any two chains has been established. To discriminate the active and passive joints, notice that

(23) $$\begin{array}{c} J_{m}\delta \Theta_{m}=J_{am}\delta \Theta_{am}+J_{pm}\delta \Theta_{pm}, \end{array}$$

where $\Theta_{am}$ and $\Theta_{pm}$ are the active and passive joint displacements of the *m*-th chain, respectively; $J_{am}$ and $J_{pm}$ are the Jacobian matrices corresponding to the active and passive joints of the *m*-th chain, respectively. In the case of the forward kinematics, the joint displacements of the actuators are known a priori, namely, $\delta \Theta_{am}=0$. Hence Equation (22) is equivalent to

(24) $$\begin{array}{c} J_{pm}\delta \Theta_{pm}-J_{pn}\delta \Theta_{pn}\approx \log^{\vee }\left(g_{n}g_{m}^{-1}\right). \end{array}$$

While Equation (24) holds for any two chains, it can be shown that only 5 of which are linearly independent. For a start, it is feasible to fix m=1 and let *n* iterate over the remaining chains. Alternatively, it is also possible to use the information of adjacent chains, which in matrix form is written as

(25) $$\begin{array}{c} J_{p}\delta \Theta_{p}\approx \delta P, \end{array}$$

where

(26) $$\begin{array}{cc} J_{p} & =\left[\begin{array}{cccccc} J_{p1} & -J_{p2} & 0 & 0 & 0 & 0\\ 0 & J_{p2} & -J_{p3} & 0 & 0 & 0\\ 0 & 0 & J_{p3} & -J_{p4} & 0 & 0\\ 0 & 0 & 0 & J_{p4} & -J_{p5} & 0\\ 0 & 0 & 0 & 0 & J_{p5} & -J_{p6} \end{array}\right], \end{array}$$

(27) $$\begin{array}{cc} \delta \Theta_{p} & ={\left[\begin{array}{cccccc} \delta \Theta_{p1}^{T} & \delta \Theta_{p2}^{T} & \delta \Theta_{p3}^{T} & \delta \Theta_{p4}^{T} & \delta \Theta_{p5}^{T} & \delta \Theta_{p6}^{T} \end{array}\right]}^{T}, \end{array}$$

(28) $$\begin{array}{cc} \delta P & ={\left[\begin{array}{ccccc} \log^{\vee }{\left(g_{2}g_{1}^{-1}\right)}^{T} & \log^{\vee }{\left(g_{3}g_{2}^{-1}\right)}^{T} & \log^{\vee }{\left(g_{4}g_{3}^{-1}\right)}^{T} & \log^{\vee }{\left(g_{5}g_{4}^{-1}\right)}^{T} & \log^{\vee }{\left(g_{6}g_{5}^{-1}\right)}^{T} \end{array}\right]}^{T}. \end{array}$$

Based on Equation (25), an algorithm for solving the forward kinematics of the general Stewart platform is summarized in Algorithm 1. When the Stewart platform is close to singularity, the small deviation in task space amounts to great joint movement, which is undesirable for real applications. To increase the robustness in the case of singularities, the algorithm uses damped least-square method in step 3. The damped least squares method, also known as the Levenberg–Marquardt method, avoids many of the pseudo-inverse method’s problems with singularities and can give a numerically stable solution. It was first used for inverse kinematics by Wampler [44] and Nakamura and Hanafusa [45].
**Algorithm 1** Forward kinematics of the general Stewart platform.**Input:**Twist coordinates of all chains, $\xi_{i}^{j},i,j=1\ldots 6$;Initial rigid displacements of all chains, $g_{i}\left(0\right),i=1\ldots 6$;Initial joint displacements of all joints for all chains, $\theta_{i}^{j}\left(0\right),i,j=1\ldots 6$;Absolute tolerance, ε;Damping factor, λ.**Output:**  Tool displacement $g_{st}$;1:Calculate tool frame pose $g_{i}$ and spatial Jacobian $J_{i}$ of each chain according to Equations (10) and (13);2:Calculate $J_{p}$ and δP according to Equations (26)–(28);3:Solve Equation (25), $\delta \Theta_{p}={\left(J_{p}^{T}J_{p}+\lambda^{2}\right)}^{-1}\delta P$;4:Update passive joint values, $\Theta_{p}=\Theta_{p}+\delta \Theta_{p}$;5:If $∥\delta \Theta_{p}∥>\varepsilon$, go to step 1;6:**return** $g_{st}$ as arbitrary $g_{i}$, i=1,2,…6.

## 4. Simulations and Experiments

In this section, simulations and experiments are conducted to demonstrate the performance of the proposed algorithm in terms of correctness, robustness, convergence ability, and execution time.

### 4.1. Simulation Setups

**Figure 3:**
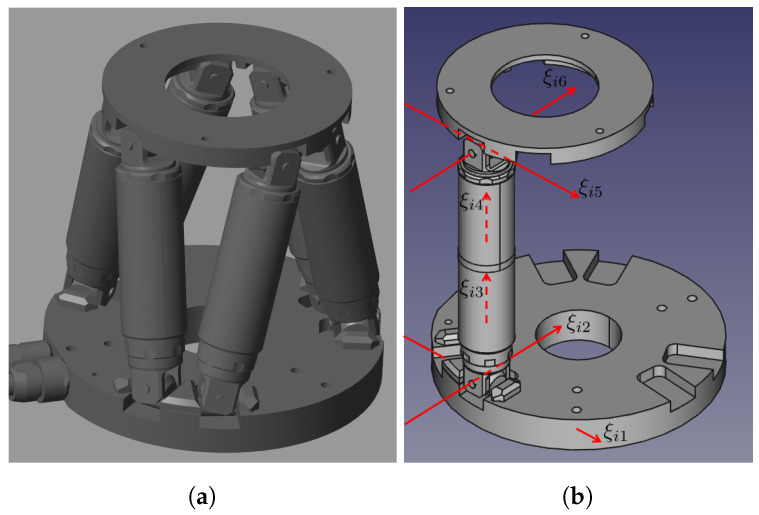
The Stewart platform: (**a**) the CAD model; (**b**) twist definitions and initial pose of the *i*-th chain.

Figure 3a shows the CAD model of the general Stewart platform used in the simulation. For each chain shown in Figure 3b, six joints can be identified, where (a) $\xi_{i1}$ and $\xi_{i2}$ are two revolute joints corresponding to the lower U-joint; (b) $\xi_{i3}$ is the linear actuation joint; (c) $\xi_{i4}$ is a screw joint; and (d) $\xi_{i5}$ and $\xi_{i6}$ are two revolute joints corresponding to the upper U-joint. Different from the traditional U-joint, the two shafts of both the lower and the upper U-joints do not intersect with each other.

**Figure 4:**
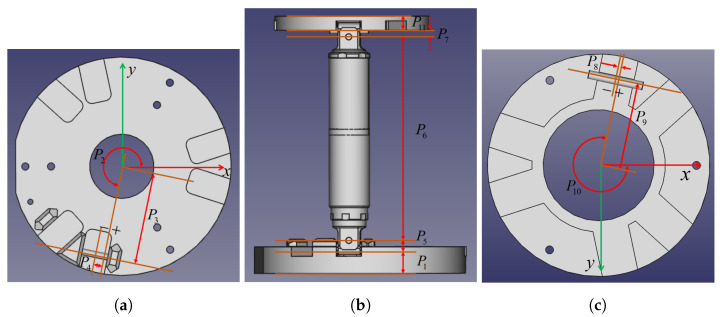
Geometric parameters for one chain: (**a**) top view of the mounting base; (**b**) side view of the linear actuator; (**c**) bottom view of the moving platform.

The home position of the *i*-th chain is also defined in Figure 3b, where (a) $\xi_{i2}$ is parallel to the base plane; (b) $\xi_{i3}$ and $\xi_{i4}$ are perpendicular to $\xi_{i1}$ and $\xi_{i2}$; (c) $\xi_{i5}$ is parallel to $\xi_{i2}$; and (d) $\xi_{i6}$ is parallel to $\xi_{i1}$. The moving platform is determined such that it is parallel to the base. As in Figure 4, to describe the initial twist coordinates and rigid displacement of the *i*-th chain, a set of 11 geometric parameters are needed. These parameters are tabulated in Table 1. The twist coordinates can then be calculated with respect to the chain kinematic parameters. The initial rigid displacement of each chain can be determined in a similar way, as in Table 2.

### 4.2. Correctness of the Proposed Algorithm

The proposed algorithm naturally solves the displacements of all passive joints, the correctness of which can therefore be verified by comparing the forward kinematics of all six chains. If the results from all chains differ within an acceptable range, the forward kinematics solution along with all the passive joint displacements constitute a valid configuration of the general Stewart platform. As there exists no bi-invariant distance between two elements $g_{0}$, $g_{1}$ of SE(3), the spatial distance and rotational distance shall be treated separately. For any two rigid body displacements $g_{0}$ and $g_{1}$,

(29) $$\begin{array}{c} \delta g=\left[\begin{array}{cccc} 0 & -\delta z & \delta y & \delta \alpha \\ \delta z & 0 & -\delta x & \delta \beta \\ -\delta y & \delta x & 0 & \delta \gamma \\ 0 & 0 & 0 & 0 \end{array}\right]=\log\left(g_{0}g_{1}^{-1}\right). \end{array}$$

The position error vector is identified as $v={\left[\begin{array}{ccc} \delta x & \delta y & \delta z \end{array}\right]}^{T}$, whose norm will be adopted as the position error. Similarly, the orientation error vector is identified as $\omega ={\left[\begin{array}{ccc} \delta \alpha & \delta \beta & \delta \gamma \end{array}\right]}^{T}$, whose norm will be used as the orientation error.

**Figure 5:**
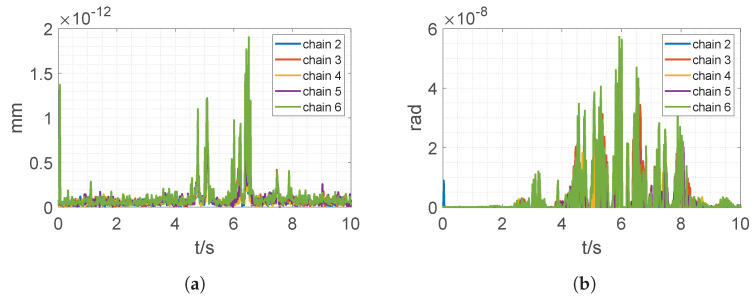
Position and orientation error compared with chain 1; (**a**) position error; (**b**) orientation error.

The standard sine wave trajectory is imposed on each actuator, i.e., $L_{i}=A_{i}\sin\left(\omega_{i}t+\phi_{i}\right)+B_{i}$. To simulate varying poses, different angular velocities are specified for each chain, as in Table 3. The motion profile lasts 10 s, with forward kinematics evaluated every 10 ms. The stopping threshold is set to $10^{-6}$, such that the maximum error will be less than $10^{-6}$ mm or $10^{-6}$ rad in all directions. Forward kinematics from the first chain is used as the return value of our algorithm, with which the results from every other chain will be compared. The resulting position and orientation errors are shown in Figure 5, where the position errors and the orientation errors are on the order of $10^{-12}$ mm and $10^{-8}$ rad, respectively, far less than the specified threshold. Hence the forward kinematic solution is accurate.

**Figure 6:**
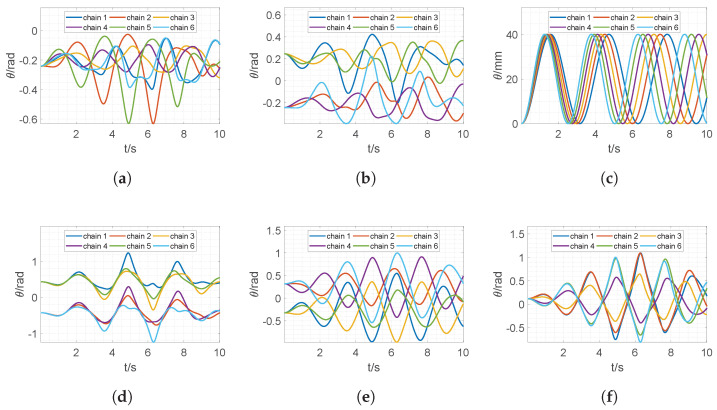
Joint displacements vs. time for all chains; (**a**) joint 1; (**b**) joint 2; (**c**) joint 3; (**d**) joint 4; (**e**) joint 5; (**f**) joint 6.

If the above solution is feasible for real applications, the trajectories of all joints should also be smooth. Figure 6 shows the trajectories of all joints during the simulation. For better visualization, joints are grouped by their orders in each chain. For example, Figure 6a plots the first joint, which is passive, of each chain, and Figure 6c corresponds to the trajectories of the active joint of each chain. Clearly, all the joint trajectories are smooth, therefore the Stewart platform transits to the final configuration smoothly.

### 4.3. Convergence and Robustness of Proposed Algorithm

To test the convergence ability and robustness of the proposed algorithm, random actuator commands are sent to the Stewart platform at home position. The maximum allowed speed of the linear actuator is around 30 mm/s, which means the joint deviation from last pose is less than 0.3 mm, assuming the cycle time being 10 ms. To test the robustness of proposed algorithm, this deviation is enlarged by a factor of 10. Randomly generated deviations uniformly distributed over [−3, 3] mm are set to all the actuators for one million times. The threshold for termination is $10^{-6}$, the same as that in the previous simulation.

**Figure 7:**
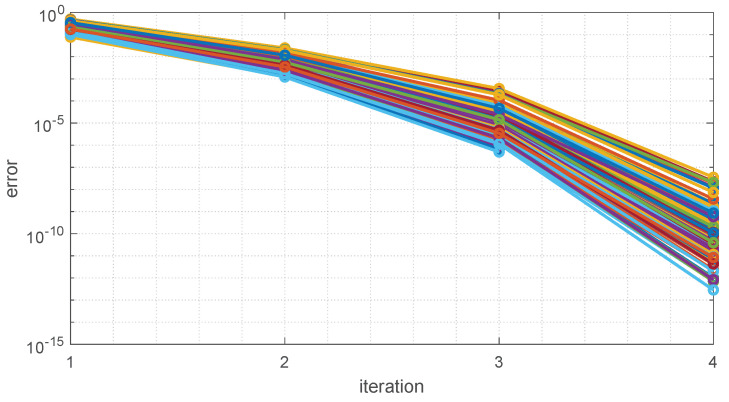
Error vs. iteration of proposed algorithm of 100 randomly selected cases.

In all test cases, the algorithm converges with maximum iteration being 4. To visualize the convergence speed, Figure 7 shows the errors at each iteration for 100 randomly selected cases. As can be seen from the figure, the error magnitude almost squared after each iteration and terminated with a maximum of 4 iterations. Therefore, the proposed algorithm possesses a near-quadratic convergence speed, which is not surprising for Levenberg–Marquardt algorithms.

### 4.4. On-Board Implementation for Real-Time Control

**Figure 8:**
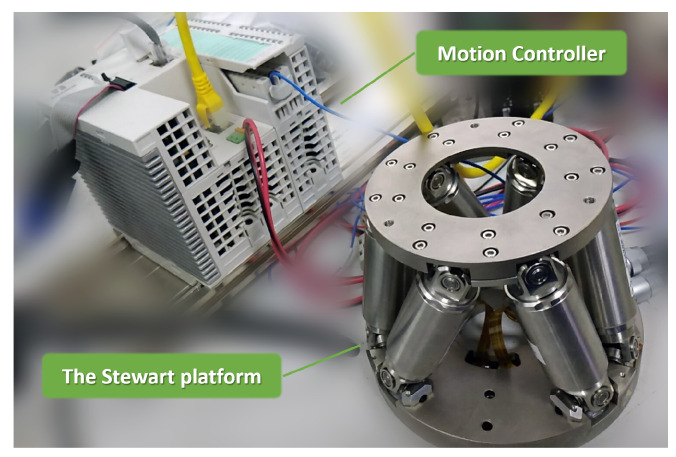
The experiment set-up.

To test the execution time in real applications, the proposed algorithm is implemented on a motion controller platform (iDEABOX 3 Pro intelligent controller), equipped with an Intel Bay Trail J1900 processor at 2.0 GHz. The cycle time of the controller is set to 10 ms. Figure 8 shows the hardware setup for our experiment. The same joint trajectory as in Section 4.2 is commanded to the controller, with termination threshold ε set to $10^{-6}$ and damping factor λ set to $10^{-8}$. The incremental method proposed in Wang [24] is implemented here as a comparison, using the same termination threshold. The maximum iteration counts for both algorithms are set to 20. There are some clarification to be made clear

1. The testing Stewart platform violates Equation (1), as it has non-intersecting upper and lower universal joints. Therefore the incremental method can’t be used. However, since $P_{5}$ and $P_{7}$ in Table 1 are quite small, in our implementation we manually set them to 0 such that the incremental method become feasible. Although such modification changes the kinematic model and is not applicable in practise, the results should still provide insights regarding the robustness and speed of the proposed algorithm.
2. To address the initial value problem, the Stewart platform is set to start at home pose for the first control cycle, namely $\Theta ={\left[\begin{array}{cccccc} 0 & 0 & 0 & 0 & 0 & 0 \end{array}\right]}^{T}$. Accordingly, the following initial end-effector displacement is used
  (30) $$\begin{array}{c} g\left(0\right)=\left[\begin{array}{cccc} 1 & 0 & 0 & 0\\ 0 & 1 & 0 & 0\\ 0 & 0 & 1 & 114.75\\ 0 & 0 & 0 & 1 \end{array}\right]. \end{array}$$
  From the second cycle and onwards, the last cycle pose is used as the initial value.

**Figure 9:**
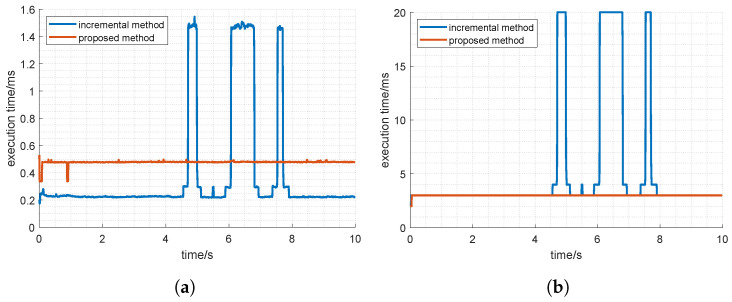
Comparison of iteration count and execution time with the incremental method; (**a**) execution time; (**b**) iteration count.

Figure 9 plots the execution times and iteration counts of both algorithms, and Table 4 summarizes some key statistics. Since the incremental method doesn’t always converge, the statistics of convergent cases are also listed.

From the results we can see that compared with the incremental method, which only applies to the 6-SPS model, the proposed algorithm is more robust, with a comparative execution time of 0.48 ms. Specifically,

1. The proposed algorithm converges at all command points, while the incremental method fails in multiple cases. Further experiment shows that the incremental method converges in all points if the magnitude of all joint trajectories decreases to 15 mm. In other words, the proposed algorithm has a larger convergent area than the incremental method, and thus is more robust. One reason that might account for the difference is that the incremental method is a Newton–Raphson method, and the proposed method belongs to Levenberg–Marquardt, which is generally considered more robust.
2. In cases where both algorithms converge, average iteration counts are 3.13 and 2.99, respectively, indicating that the two algorithms share basically the same convergence ability. This can be explained as the Levenberg–Marquardt degenerating to a Newton–Raphson when using a very small damping factor.
3. In case the incremental method converges, the execution time is around half of the proposed method’s. Since the iteration counts for two algorithms are almost identical, we conclude that the complexity of the iteration equation leads to the difference. Indeed, although $J_{p}$ is quite sparse, it is a 30-by-30 matrix, whereas the counterpart in the incremental method is 6-by-6. To this point, we would like to mention that the extra complexity is not for nothing. For one thing, the incremental method is constructed for the ideal 6-SPS platform, so it can’t be applied directly to other variants such as the 6-RSS. Moreover, subject to manufacturing ability, a calibration process is usually needed to increase the accuracy. For this purpose, an error model is indispensable, which might break Equation (1) by considering imperfect ball joints. The incremental method is infeasible in both cases. Whereas the proposed method can still be adopted by simply modifying the corresponding twist coordinates.

## 5. Conclusions

In this paper, a geometric algorithm was proposed for real-time forward kinematics of the general Stewart platform, where no geometric constraints, such as perpendicularity, parallelism, and intersection, are presumed between joints. Specifically, a geometric approach, which describes the rigid body motion as exponential mapping of twist was used to formulate the problem. A very neat and simple differential relationship between task space and joint space was therefore established. The proposed algorithm is derived for the general Stewart platform, and can work for other existing spatial manipulators, including but not limited to 6-RUS and 6-RSS. Experiments and simulations were carried out to demonstrate the effectiveness of the proposed algorithm. The results showed that the proposed algorithm is accurate, robust, and fast. In all test cases, the proposed algorithm terminates with maximum iteration being 4 and converges with near-quadratic speed. The proposed algorithm is also implemented on an embedded controller. Compared with the incremental method, which only applies to the 6-SPS model, the proposed algorithm is more robust, with a comparative execution time of 0.48 ms. Overall, the efficiency of proposed algorithm meets the requirements of most applications, such as kinematic calibration, motion simulation, and real-time control. Some of the future work are summarized as follows.

1. As the damping factor λ has great influence on the efficiency of the Levenberg–Marquardt method, instead of using a fixed damping factor, a more effective strategy can be explored considering manipulability and other factors.
2. Calibration can greatly increase the accuracy of the end-effector. Most existing algorithms considered the errors from actuator lengths, ball joint location, and motion errors; developing a calibration model that applies to the general Stewart platform is necessary.

## Figures and Tables

**Table 1:** Geometric parameters of all chains.

	$P_{1}$	$P_{2}$	$P_{3}$	$P_{4}$	$P_{5}$	$P_{6}$	$P_{7}$	$P_{8}$	$P_{9}$	$P_{10}$	$P_{11}$
	(mm)	(deg)	(mm)	(mm)	(mm)	(mm)	(mm)	(mm)	(mm)	(deg)	(mm)
chain 1	17.00	18.00	57.00	−5.06	4.20	85.00	4.20	0.56	39.00	42.00	9.00
chain 2	17.00	102.00	57.00	5.06	4.20	85.00	4.20	−0.56	39.00	78.00	9.00
chain 3	17.00	138.00	57.00	−5.06	4.20	85.00	4.20	0.56	39.00	162.00	9.00
chain 4	17.00	222.00	57.00	5.06	4.20	85.00	4.20	−0.56	39.00	198.00	9.00
chain 5	17.00	258.00	57.00	−5.06	4.20	85.00	4.20	0.56	39.00	282.00	9.00
chain 6	17.00	342.00	57.00	5.06	4.20	85.00	4.20	−0.56	39.00	318.00	9.00

**Table 2:** Initial rigid displacement of each chain.

	Translation			Orientation
	*x* (mm)	*y* (mm)	*z* (mm)	(deg)
chain 1	18.51	1.28	119.40	Rotz(−24.00)
chain 2	−8.14	16.67	119.40	Rotz( 24.00)
chain 3	−10.37	15.39	119.40	Rotz(−24.00)
chain 4	−10.37	−15.39	119.40	Rotz( 24.00)
chain 5	−8.14	−16.67	119.40	Rotz(−24.00)
chain 6	18.51	1.28	119.40	Rotz( 24.00)

**Table 3:** Sine trajectory parameters of each chain.

	$A_{i}$	$\omega_{i}$	ϕ	$B_{i}$
	(mm)	(rad/s)	(rad)	(mm)
chain 1	20.00	2.00	0.00	20.00
chain 2	20.00	2.10	0.00	20.00
chain 3	20.00	2.20	0.00	20.00
chain 4	20.00	2.30	0.00	20.00
chain 5	20.00	2.40	0.00	20.00
chain 6	20.00	2.50	0.00	20.00

**Table 4:** Statistics of forward kinematics of two methods.

	Average Iteration Count	Max Iteration Count	Average Execution Time	Max Execution Time
incremental method (all cases)	5.12	20	0.38	1.54
incremental method (convergent cases)	3.13	17	0.23	1.25
proposed method	2.99	3	0.48	0.53

## Data Availability

Not applicable.
